# P-1805. Novel Cytomegalovirus Mutations in Immunocompromised Patients: Genotypic Insights and Clinical Correlation

**DOI:** 10.1093/ofid/ofaf695.1974

**Published:** 2026-01-11

**Authors:** Abdulrahman M AlSweed, Madain S Alsanea, Fatimah AlHamlan, Ahmed Alqahtani, Reem AlMaghrabi

**Affiliations:** King Faisal Specialist Hospital & Research Center, Riyadh, Ar Riyad, Saudi Arabia; King Faisal Specialist Hospital & Research Center, Riyadh, Ar Riyad, Saudi Arabia; King Faisal Specialist Hospital & Research Center, Riyadh, Ar Riyad, Saudi Arabia; King Faisal Specialist Hospital & Research Center, Riyadh, Ar Riyad, Saudi Arabia; King Faisal Specialist Hospital and Research Center, Riyadh, Ar Riyad, Saudi Arabia

## Abstract

**Background:**

Cytomegalovirus (CMV) is a substantial cause of morbidity in immunocompromised populations, particularly among solid organ and bone marrow transplant recipients. The emergence of novel mutations, particularly within conserved genomic regions, presents challenges for interpreting genotypic resistance and guiding therapy. The aim is to support the correlation of clinical variability with known genotypic resistance, enhance the understanding of viral behavior among different host factors that play the utmost role in managing CMV infection, and investigate the potential impact of the novel mutations.

**Figure:**
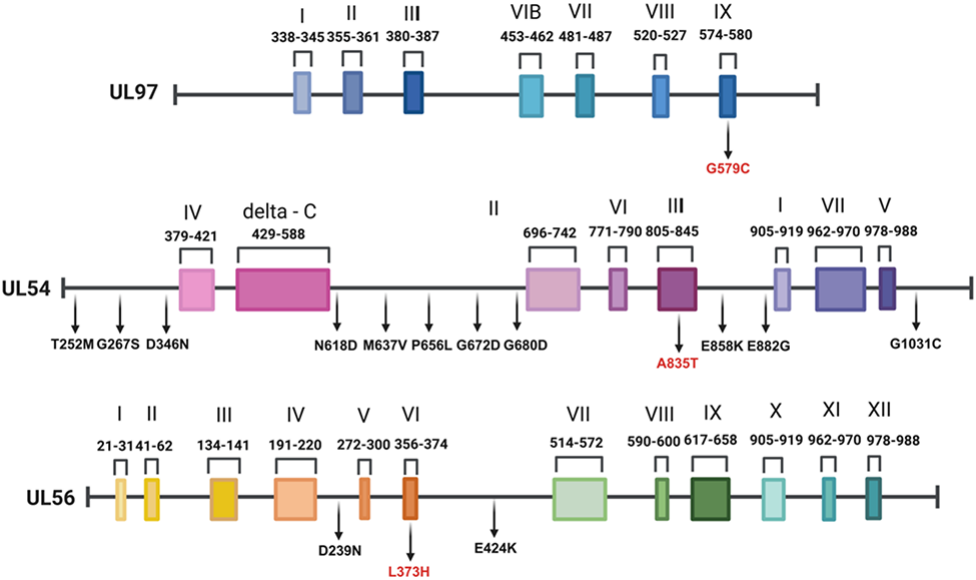
Novel CMV mutations found in UL54, UL97, and UL56 aligned along conserved regions The figure illustrates conserved regions among UL54, UL97, and UL56 genes. The novel mutations marked in red align along conserved regions.

**Figure:**
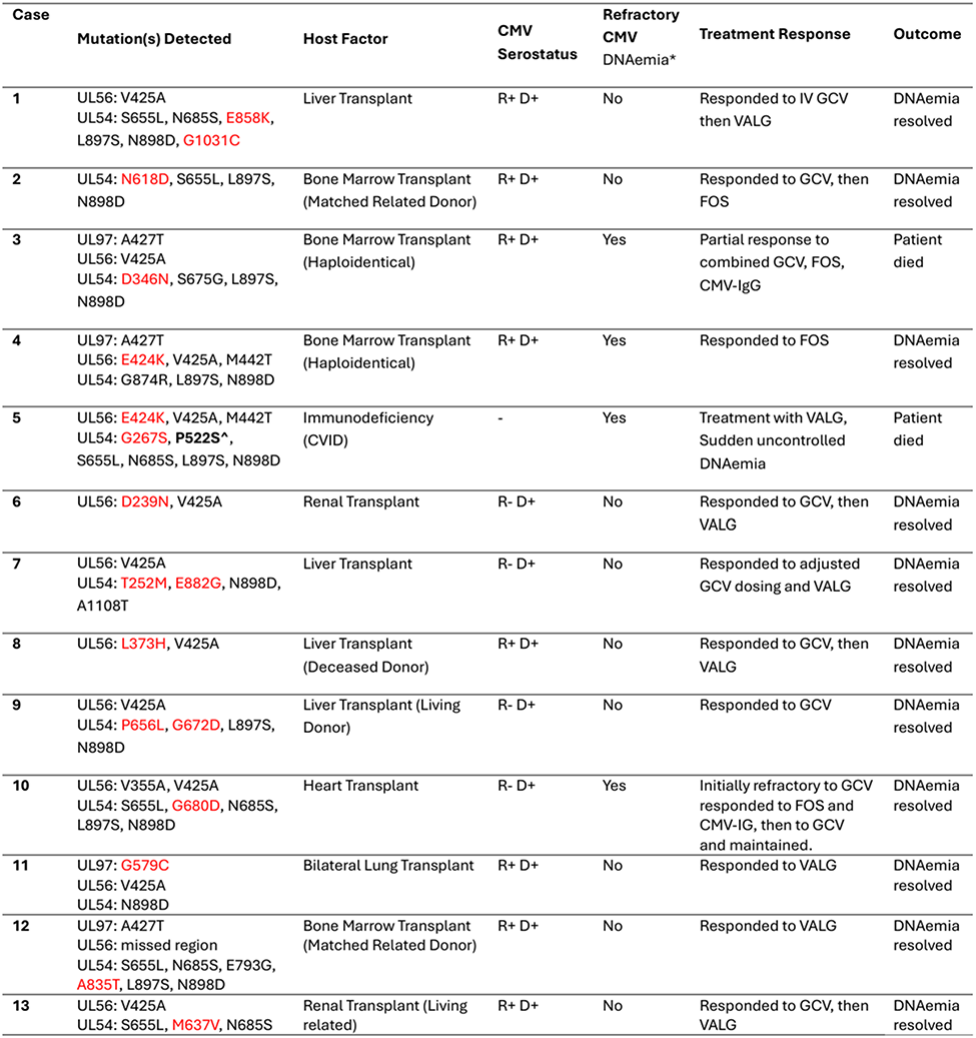
Cases summary with novel CMV mutations The table shows patients with novel CMV mutations, their background, clinical antiviral course, and outcomes. *Refractory CMV infection: Increase by > 1 log10 CMV DNA levels in blood or plasma after at least 2 weeks of appropriately dosed anti-CMV medication. Abbreviations: GCV: Ganciclovir, VALG: Valganciclovir, FOS: Foscarnet, R: recipient of transplanted organ/bone marrow, D: donor. Reported mutations are natural polymorphisms except for marked mutations; bold indicates a known drug resistance mutation; red indicates a novel mutation. ^IC50 for GCV was reported in the literature as 3.1 (intermediate level)

**Methods:**

Fifty-one CMV-positive clinical specimens from immunocompromised patients were subjected to UL97, UL54, and UL56 sequencing. Variants were aligned to reference genomes (Merlin strain) and assessed via BLAST for novelty and conservation. Clinical outcomes, antiviral regimens, and virologic responses were reviewed retrospectively.

**Results:**

Thirteen patients exhibited previously unreported mutations in UL54, UL97, and UL56. Although four cases met the criteria for refractory DNAemia, some have responded favorably to first-line antivirals. Among the mutations analyzed, G579C in UL97 and A835T in UL54 were identified in conserved regions essential for substrate binding and polymerase activity, raising concern for functional relevance. One case harbored UL54 P522S, a known mutation associated with intermediate ganciclovir resistance. Two patients with profound immunosuppression and refractory DNAemia died, highlighting the critical influence of host factors on clinical outcomes.

**Conclusion:**

CMV drug resistance mutations (DRMs) must be analyzed cautiously, as host response can be the main determining factor for DNAemia clearance. Early and specific DRM reporting is crucial, especially in immunocompromised hosts; hence, genotyping is the best modality. However, we suggest interpreting these findings according to the clinical response and reported known recombinant phenotypic testing (EC50/IC50). The additional benefit of classification for DRMs to low, intermediate, or high can aid physicians in deciding about medication switch versus dose adjustment of ongoing antiviral therapy, especially in the initial response to treatment.

**Disclosures:**

All Authors: No reported disclosures

